# Ultrasound assessment of gastric contents and volume in patients undergoing endoscopic endonasal transsphenoidal surgery: a prospective observational study

**DOI:** 10.1038/s41598-023-29893-2

**Published:** 2023-02-17

**Authors:** Haitao Jia, Ertao He, Shixiong Gao, Wei Hao, Yanli Li, Wei Liu, Xiaoxia Chen, Yanfei Jia, Yingbin Wang

**Affiliations:** 1 Department of Anesthesiology and Postanesthesia Care Unit, Lanzhou University Second Hospital, Chengguan District, Lanzhou, 730030 Gansu China; 2 Department of Neurosurgery, Lanzhou University Second Hospital, Lanzhou, 730030 Gansu China

**Keywords:** Neuroscience, Diseases, Endocrinology, Gastroenterology, Health care, Medical research, Neurology, Oncology, Risk factors

## Abstract

Intraoperative ingestion of blood, cerebrospinal fluid, and irrigation fluid can lead to an increase in gastric volume, resulting in the potential risk of aspiration in patients after endoscopic endonasal transsphenoidal surgery (EETS). In this prospective observational study, we aimed to assess the volume of gastric contents in patients undergoing this neurosurgical procedure using ultrasound, and to determine the factors associated with volume change. Eighty-two patients diagnosed with pituitary adenoma were recruited consecutively. Semi-quantitative (Perlas scores: 0, 1 and 2) and quantitative (cross-sectional area, CSA) ultrasound assessments of the gastric antrum were performed immediately before and after surgery in the semi-recumbent and semi-recumbent right-lateral positions. Seven (8.5%) patients had antrum scores from preoperative grade 0 to postoperative grade 2; nine (11%) patients had antrum scores from preoperative grade 0 to postoperative grade 1. The mean ± standard deviation (SD) of increased gastric volume was 71.0 ± 33.1 mL and 236.5 ± 32.4 mL in postoperative grade 1 and 2 groups, respectively. Subgroup analysis showed that 11 (13.4%) patients (4 in grade 1 and all in grade 2) had postoperative estimated gastric volume > 1.5 mL kg^−1^ (mean ± SD 3.08 ± 1.67, range 1.51–5.01 mL kg^−1^). Logistic regression analysis revealed that older age, diabetes mellitus, and long surgical duration were independent risk factors for significant volume change (all *P* < 0.05). Our results showed a significant increase in gastric volume in some patients who underwent EETS. Bedside ultrasound measurements of gastric volume can be used to assess the postoperative aspiration risk, particularly in older diabetic patients with a longer surgical duration.

## Introduction

Perioperative aspiration of gastric contents is a serious pulmonary complication associated with a significant morbidity and mortality rate of 5–9% in anesthesia airway management accidents^1–5^. Although aspiration often occurs at anesthesia induction in patients with passive regurgitation, recovery from anesthesia and tracheal extubation are also risk periods for aspiration events in patients with active vomiting^6^. The presence of a large volume of gastric contents is one of the most significant risk factors for pulmonary aspiration during anesthesia management^7^.

Endoscopic endonasal transsphenoidal surgery (EETS) comprises a large proportion of neurosurgical surgeries and is widely used to resect pituitary tumors^8^. Patients undergoing EETS have a significant incidence of postoperative nausea and vomiting (PONV) due to leakage of cerebrospinal fluid and fat grafting^9^. In addition, during this special surgical procedure, ingestion of blood, cerebrospinal fluid, and irrigation fluid can lead to a significant increase in gastric volume^10^. Ingested blood is generally considered a potent emetic that can also lead to frequent postoperative vomiting^10,11^. All these conditions result in a potential postoperative risk of aspiration in patients during recovery of consciousness and protective upper airway reflex. Several medical measures are usually taken to treat postoperative vomiting and prevent pulmonary aspiration in this surgical population^12^. However, the incidence and related factors of a significant increase in intraoperative gastric volume and its clinical significance in patient outcomes are unclear. In this study, we aimed to assess the gastric contents and volume in patients undergoing this neurosurgical procedure using bedside ultrasound, and to determine the patients and surgical factors associated with intraoperative volume change.

We hypothesized that significant changes in gastric volume occur in some patients during EETS. The primary outcomes were antral grade before and after transsphenoidal surgery. Secondary outcomes included antral CSA, gastric volume, and incidence and factors associated with significant volume increase.

## Materials and methods

### Study design and approval

This prospective observational study was registered at http://www.chictr.org.cn (ChiCTR2100045110) on April 7, 2021, and approved by the Institutional Ethics Committee of Lanzhou University Second Hospital (Lanzhou, China) on May 11, 2021 (protocol number 2021A-381). Informed consent was obtained from each participant before study initiation. This study was designed and conducted in accordance with the Guidelines on Strengthening the Reporting of Observational Studies in Epidemiology and the Helsinki Declaration.

This study was conducted at a tertiary teaching hospital between May 12, 2021 and September 30, 2022. The inclusion criteria were as follows: (i) patients aged 18–80, (ii) American Society of Anesthesiologists (ASA) physical status II–III, (iii) diagnosis of pituitary adenoma, and (iv) elective EETS for pituitary tumor excision. Exclusion criteria were: (i) body mass index (BMI) ≥ 35 kg m^−2^, (ii) altered state of consciousness, (iii) pregnancy, (iv) patients with abnormal anatomy of the upper gastrointestinal tract (including previous esophageal or gastric surgery, stricture and tumors), (v) failure to follow preoperative fasting guidelines, (vi) hemodynamic instability, and (vii) transfer to the intensive care unit after surgery. According to our hospital's institutional practice standards, all patients fasted for at least 8 h for solid foods (bread, rice, and noodles) and 2 h for clear fluids (carbohydrate beverages, tea, and fruit juice) before elective surgery. No antacids or prokinetic agents were administered prophylactically.

### Gastric ultrasonography

Upon arrival in the operating room, the participants were placed in semi-recumbent and semi-recumbent right lateral positions, with the head of the bed elevated 30° for preoperative ultrasound scanning. We used a portable ultrasound device with a curved low-frequency (2–5 MHz) probe and high-resolution transducer (FUJIFILM SonoSite Bothell, Washington, USA). We followed the standardized gastric ultrasound scanning protocol as previously described^13^. Briefly, the ultrasonic probe was placed on the epigastria to obtain a sagittal scanning plane. To locate the gastric antrum, we used the left lobe of the liver and the posterior pancreas as internal anatomical landmarks on the plane of the inferior vena cava.

We initially assessed the nature (nil, fluid, or solid particles) of the gastric contents. An empty stomach appeared as the antrum walls juxtaposed with a "flat" appearance. The clear fluid appeared as "homogeneously hypoechoic" liquid content without residues. The solid contents appeared as a "frosted glass" accompanied by acoustic shading, which disturbed the visualization of the posterior wall^13^. Semi-quantitative assessment of the antrum was carried out in the semi-recumbent and semi-recumbent right lateral positions according to the three-point grading system (Perlas score)^14^: grade 0 corresponded to the absence of any content in both positions (complete empty stomach); grade 1 corresponded to the visualization of liquid content only in the semi-recumbent right lateral position (low volume status); and grade 2 corresponded to the visualization of liquid content in both positions (high volume status).

Quantitative assessment of gastric contents was carried out only in the semi-recumbent right lateral position. The antral CSA (cm^2^) was obtained using the free-tracing caliper of the ultrasound machine and included the entire serous layer. We used the mean of three consecutive measurements of this area during the interval of peristaltic contractions for the final analysis. Estimated gastric volumes were calculated using a previously validated formula: gastric volume (mL) = 27.0 + (14.6 × CSA) – (1.28 × Age)^15^. Based on previous literature^16^, we defined ‘Risk Stomach’ as the antrum score was grade 2 regardless of volume and grade 1 with gastric volume > 1.5 mL kg^−1^, while ‘Non-risk Stomach’ was grade 0 and grade 1 with gastric volume ≤ 1.5 mL kg^−1^.

Following preoperative ultrasonography, all participants were placed in a supine position and continuously monitored with electrocardiography, peripheral oxygen saturation, and noninvasive blood pressure measurements. All the patients were premedicated with intravenous midazolam (0.05 mg kg^−1^). General anesthesia was intravenously induced with etomidate (2 mg kg^−1^) and sufentanil (0.5 μg kg^−1^), followed by tracheal intubation with rocuronium (0.6 mg kg^−1^). Anesthesia was maintained by intravenous administration of propofol (4–6 mg kg^−1^ h^−1^) or inhalation anesthesia (2% sevoflurane in 50% oxygen and air mixtures) and continuous intravenous administration of remifentanil (0.2–0.3 μg kg^−1^ min^−1^). We continuously monitored the depth of anesthesia using a bi-spectral index and targeted the range of 40–60. All patients received an intravenous injection of 8–16 mg ondansetron near the end of the operation to prevent PONV.

An experienced neurosurgical team performed the EETS for pituitary tumor resection using standard surgical procedures^8^. All patients were placed in a semi-recumbent position with the head of the bed elevated to 15–20° and their heads slightly turned towards the surgeon. After the nasal cavity was enlarged, the sellar floor was dissected to expose the pituitary fossa and the tumor was curetted. The sellar cavity was then packed with gelatin sponges or fat grafts for hemostasis, and laid over using dural substitutes. Following revision hemostasis, intranasal anatomy was restored without nasal packing. The endoscope lens and operating field were continuously cleaned throughout the surgical procedure by using an irrigation sheath connected to an automated irrigation system. The irrigation fluid (0.9% saline) was suctioned intermittently using a negative pressure suction device.

After surgery, all patients immediately underwent postoperative gastric ultrasonography, similar to the protocol for preoperative scanning positions for semi-quantitative and quantitative evaluation. The patients were then transferred to the postanesthesia care unit for recovery from anesthesia and tracheal extubation. If necessary, 30 mg ketorolac was administered intravenously for postoperative pain management. We recorded the incidence of regurgitation or vomiting, and pulmonary aspiration within 2 h after surgery.

### Data collected

Preoperative ultrasonography was performed by an attending anesthesiologist (HT.J), and postoperative ultrasonography was performed by another anesthesiologist (ET.H) who was blinded to the preoperative findings and surgical procedures. Each of them had performed at least 50 gastric ultrasound examinations on adult patients. The correlation coefficient of intra- and inter-rater reliability of the antral measurements was > 0.92 in our pilot trial. Another anesthesiologist collected and analyzed the data.

The following patient characteristics and surgical variables were collected: sex, age, weight, height, body mass index, ASA physical status classification, fasting duration for solids and liquids, anesthesia method, classification of pituitary tumors, duration of anesthesia and surgery, intraoperative volumes of irrigation fluid and blood loss, and the status of comorbidities such as hypertension, diabetes mellitus, esophageal motility disorders (achalasia, diffuse esophageal spasm), gastroesophageal reflux, and hiatal hernia.

### Statistical analysis

We performed the Kolmogorov–Smirnov test to assess continuous variables with a normal distribution. Normally distributed continuous variables were expressed as mean ± standard deviation (SD) and compared using Student’s *t*-test. Non-normally distributed continuous variables were expressed as median (interquartile [range]) and compared using the Wilcoxon signed-rank test. Categorical variables were expressed as frequencies (percentage) or ratios and compared using Pearson’s chi-square test. One-way ANOVA was used for multiple comparisons to evaluate the differences in antral CSAs and corresponding gastric volumes between different antral grades after transsphenoidal surgery. In addition, we used univariate and multivariate binary logistic regression analysis to identify the patients and surgical factors associated with postoperative risk stomach. All significant variables (*P* < 0.10) associated with postoperative risk stomach in univariate logistic regression analysis were subjected to multivariate analysis using a stepwise method. The Hosmer–Lemeshow test was used to determine the goodness of fit of multivariate logistic regression analysis models with *P* > 0.05. The results are presented as odds ratios (OR) with 95% confidence intervals (*CI*). Statistical analyses were performed using SPSS version 19.0 (IBM Armonk, New York, USA). Differences were considered statistically significant at *P* < 0.05, using a two-tailed test.

## Results

**Figure 1:**
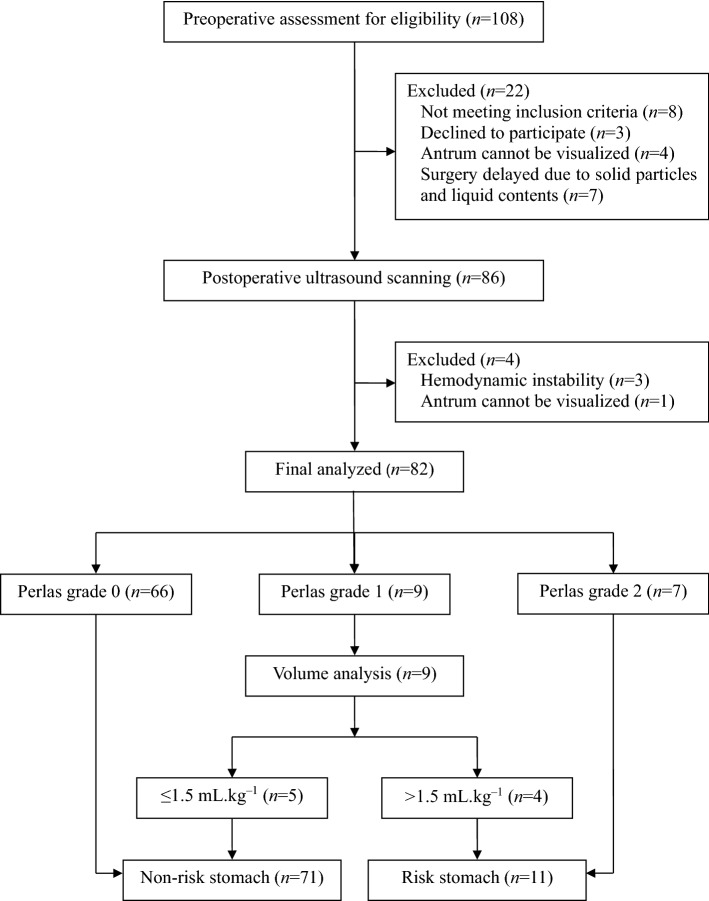
Patient flow and analysis chart.

A total of 108 patients were enrolled consecutively. We excluded eight patients who did not meet the inclusion criteria and three who were unwilling to participate in the eligibility assessment. After preoperative ultrasound screening, seven patients underwent delayed surgery due to the presence of solid particles or liquid gastric contents, and the antrum in four patients could not be clearly visualized. In addition, three patients had hemodynamic instability and the antrum in one patient could not be clearly visualized on postoperative ultrasound scanning. The remaining 82 patients with preoperative empty stomach (Perlas grade 0) completed the surgery as planned and were included in the final analysis (Fig. 1). Table 1 presents the demographic characteristics and other baseline variables. The mean ± SD of fasting duration was 8.7 ± 1.9 h and 3.7 ± 1.4 h for solid foods and clear liquids, respectively. The most common type of pituitary adenoma in our cohort was non-functioning (56%), followed by lactotrophs (17%), corticotrophs (11%), somatotrophs (10%), and thyrotrophs (6%). Forty-five (54.9%) and 37 (45.1%) patients received total intravenous and inhalation anesthesia, respectively.

**Table 1:** Baseline characteristics of the participants included in final analysis.

Characteristics	Frequency
Sex	
Male	44 (53.7%)
Female	38 (46.3%)
Age (years)	52 ± 14
Weight (kg)	66.2 ± 7.8
Height (cm)	165.8 ± 8.7
Body mass index (kg m^−2^)	24.2 ± 3.5
ASA physical status	
II	64 (78%)
III	18 (22%)
Fasting duration for solids (h)	8.7 ± 1.9
Fasting duration for liquids (h)	3.7 ± 1.4

Values are presented as n (%) or mean ± SD.

**Figure 2:**
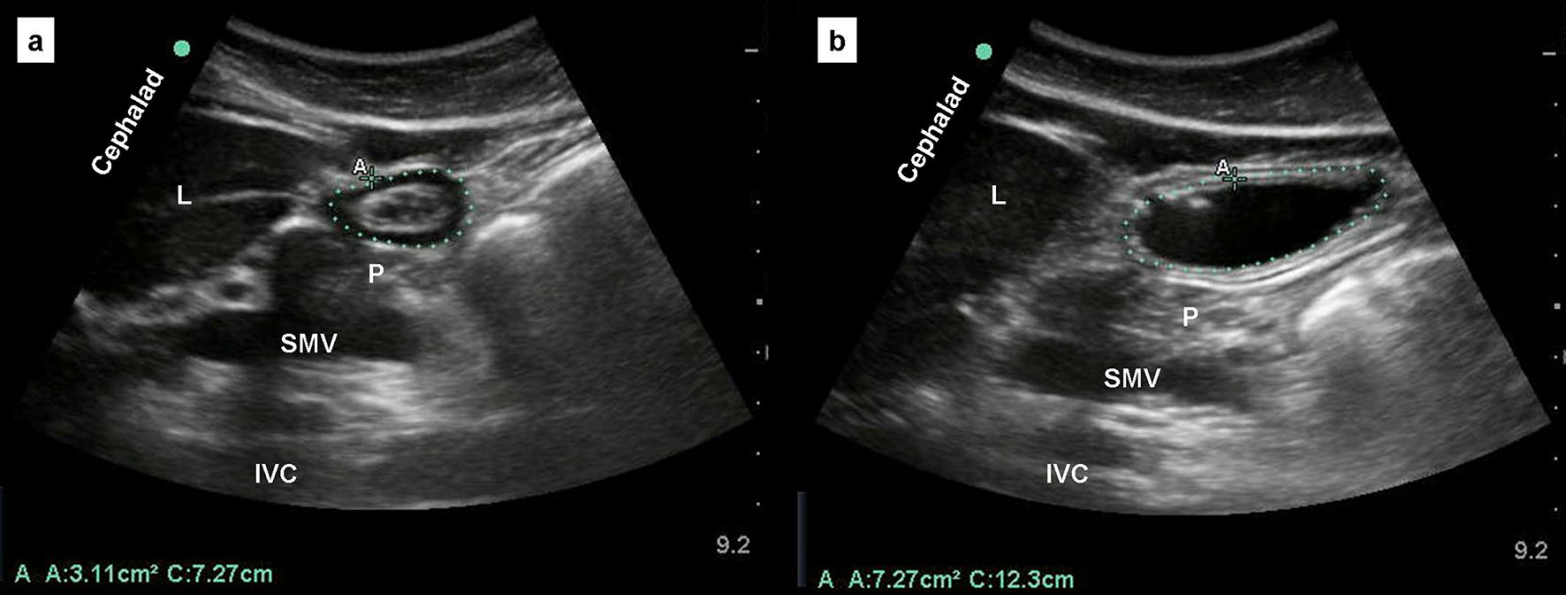
Antral ultrasound views from the same patient in the semirecumbent position (**a**) before and (**b**) after endoscopic endonasal transsphenoidal pituitary surgery. L: liver; P: pancreas; SMV: superior mesenteric vein; IVC: inferior vena cava. Dotted circle: gastric antrum.

Table 2 summarizes the results of semi-quantitative and quantitative ultrasound assessments of gastric contents before and after transsphenoidal surgery. Of the 82 patients, seven (8.5%) had an antrum score from preoperative grade 0 to postoperative grade 2, nine (11%) from preoperative grade 0 to postoperative grade 1, and the remaining 66 (80.5%) showed no changes in gastric contents after surgery (grade 0). There were significant differences in postoperative CSAs and estimated gastric volumes between grades (all *P* < 0.05). The mean ± SD of increased gastric volume was 71.0 ± 33.1 mL and 236.5 ± 32.4 mL in the postoperative grade 1 and grade 2 groups, respectively. Figure 2 depicts a gastric ultrasound image of the same patient that shows an empty stomach (flat antrum) before (A) and a large volume of liquid gastric contents (homogeneously hypoechoic) after (B) transsphenoidal surgery.

**Table 2:** Antral cross-sectional areas and estimated gastric volumes between different antrum grades before and after transsphenoidal surgery.

Parameters	Before surgery	After surgery			*P*-value
	Grade 0 (*n* = 82)	Grade 0 (*n* = 66)	Grade 1 (*n* = 9)	Grade 2 (*n* = 7)	
Semi-recumbent position					
Antral cross-sectional area (cm^2^)	3.21 ± 0.24	3.27 ± 0.19	3.44 ± 0.26	9.54 ± 1.12	0.001
Semi-recumbent right lateral position					
Antral cross-sectional area (cm^2^)	3.32 ± 0.23	3.32 ± 0.14	8.13 ± 0.17	20.24 ± 1.75	0.001
Estimated gastric volume (mL)	14.15 ± 12.46	14.71 ± 14.29	71.57 ± 23.77	236.58 ± 30.09	0.001
Estimated gastric volume (mL kg^−1^)	0.22 ± 0.19	0.23 ± 0.22	1.08 ± 0.43	3.89 ± 0.56	0.001

Values are presented as mean ± SD.

To identify the risk factors associated with intraoperative volume increase, we further categorized patients into the risk stomach group (> 1.5 mL kg^−1^) and non-risk stomach group (≤ 1.5 mL kg^−1^). In this subgroup analysis, we found that 11 (13.4%) patients (4 in grade 1 and all in grade 2) had postoperative gastric volume > 1.5 mL kg^−1^ (mean ± SD 3.08 ± 1.67, range 1.51–5.01 mL kg^−1^). The baseline characteristics and surgical variables of patients in the different groups are shown in Table 3. The results of logistic regression analysis revealed that older age (odds ratio 1.186, 95% *CI* 1.046–1.346), diabetes mellitus (odds ratio 11.016, 95% *CI* 1.238–98.026), and long surgical duration (odds ratio 1.028, 95% *CI* 1.008–1.048) were independent risk factors for postoperative risk stomach and were associated with significant intraoperative volume changes (all *P* < 0.05) (Table 4).

**Table 3:** The baseline characteristics and surgical variables of patients with postoperative risk stomach and non-risk stomach. Values are presented as mean ± SD, median (interquartile [range]) or n (%).

Variable	Non-risk stomach group (*n* = 71)	Risk stomach group (*n* = 11)	*P*-value
Age (years)	50.0 ± 13.6	65.1 ± 8.4	0.001
Sex, male:female	39 (54.9%):32 (45.1%)	6 (54.5%):5 (45.5%)	0.981
Body mass index (kg m^−2^)	24.6 ± 3.5	22.2 ± 2.6	0.034
ASA physical status II:III	57 (80.3%):14 (19.7%)	7 (63.6%):4 (36.4%)	0.396
Fasting duration for solids (h)	8.6 ± 1.9	9.1 ± 2.0	0.449
Fasting duration for fluids (h)	3.8 ± 1.4	3.5 ± 1.3	0.477
Intravenous:inhalation anesthesia	39 (54.9%):32 (45.1%)	6 (54.5%):5 (45.5%)	0.981
Anesthesia duration (min)	242.9 ± 65.8	300.9 ± 76.3	0.009
Operation duration (min)	221.6 ± 64.9	268.2 ± 80.4	0.035
Volume of irrigation (ml)	1300 (800, 2600 [50, 6500])	2400 (800, 4000 [200, 8000])	0.316
Total blood loss (ml)	260 (100, 400 [40, 800])	360 (250, 650 [100, 1050])	0.431
Non-functioning:functioning adenomas	49 (69.0%):22 (31.0%)	7 (63.6%):4 (36.4%)	0.993
Preoperative hypertension	20 (28.2%)	6 (54.5%)	0.161
Diabetes mellitus	12 (16.9%)	8 (72.7%)	0.000
Oesophageal motility disorders	4 (5.6%)	2 (18.2%)	0.182
Gastroesophageal reflux	5 (7.1%)	2 (18.1%)	0.236
Hiatal hernia	3 (4.2%)	1 (9.1%)	0.444

**Table 4:** Logistic regression analyses of the factors associated with postoperative risk stomach (> 1.5 mL kg^−1^).

Variable	Univariate analysis		Multivariate analysis	
	*P*-value	Odds ratio (95% *CI*)	*P*-value	Odds ratio (95% *CI*)
Sex	0.949	1.042 (0.291–3.730)		
Age	0.003	1.113 (1.038–1.193)	0.008	1.186 (1.046–1.346)
Body mass index	0.043	0.762 (0.586–0.991)		
ASA physical status classification	0.196	3.250 (0.545–19.383)		
Type of anesthesia	0.773	1.207 (0.336–4.331)		
Anesthesia duration	0.016	1.012 (1.002–1.021)		
Operation duration	0.003	1.024 (1.010–1.037)	0.005	1.028 (1.008–1.048)
Volume of irrigation fluid	0.077	1.001 (1.000–1.001)		
Blood loss	0.654	1.001 (0.997–1.004)		
Esophageal motility disorders	0.324	2.407 (0.420–13.792)		
Gastroesophageal reflux	0.002	11.003 (2.467–49.053)		
Hiatal hernia	0.809	1.320 (0.139–12.495)		
Preoperative hypertension	0.192	2.350 (0.650–8.492)		
Diabetes mellitus	0.003	7.808 (1.988–30.658)	0.031	11.016 (1.238–98.026)
Fasting duration for solids	0.445	1.136 (0.819–1.577)		
Fasting duration for liquids	0.473	0.842 (0.522–1.353)		

All patients with significant postoperative volume changes (antrum grade 2 and 1) underwent orogastric suctioning under ultrasound guidance. Nine patients had PONV, and none experienced aspiration or reintubation after tracheal extubation.

## Discussion

In this prospective observational study, we used ultrasound to assess the gastric contents and volume in patients before and after elective endoscopic endonasal transsphenoidal pituitary surgery. As the most important finding of this study, the results of the semi-quantitative analysis showed that some patients had significant changes in gastric volume after surgery. Seven (8.5%) patients had changes in the antrum score from preoperative grade 0 to postoperative grade 2, while nine (11%) patients had changes from preoperative grade 0 to postoperative grade 1. Further quantitative analysis showed that 11 (13.4%) patients had a postoperative estimated gastric volume more than 1.5 mL kg^−1^, which was significantly larger than that expected in fasting adult patients who underwent elective surgical procedures. Obviously, this change predisposes these neurosurgical patients with significant PONV to a high risk of aspiration during recovery from anesthesia and extubation when swallowing function and protective airway reflex are not fully recovered.

Perioperative aspiration and subsequent pneumonia can result in hypoxemia, longer mechanical ventilation, longer hospital stays, higher hospital charges, and increased mortality^17^. Previously, assessment of perioperative aspiration risk depended almost exclusively on the duration of preoperative fasting. Current practice guidelines suggest that fasting for at least 6 h after a light meal and 2 h after a clear liquid before induction of anesthesia is adequate to empty gastric contents and minimize the perioperative aspiration risk in adult patients undergoing elective surgery^7^. However, these guidelines are not applicable to patients with certain critical comorbidities and cannot predict changes in the gastric contents during surgical procedures. Some surgical procedures may affect intraoperative changes in the gastric content and volume. Therefore, immediate measurement of the gastric contents and volume during anesthesia management is essential to assess the potential risk of aspiration.

EETS is a neurosurgical procedure that is widely used to resect pituitary tumors. During this special surgical procedure, the head of the bed was elevated by 15–20° (reverse Trendelenberg position) to reduce bleeding and cerebrospinal fluid leakage. However, this position potentially allows downward flow of blood, cerebrospinal fluid, and irrigation fluid (used to clean the endoscope lens and operating field) from the nasopharynx to the esophagus and stomach due to gravity^8,10^. We used bedside ultrasound to measure gastric volume in patients after EETS, and guided orogastric suction in patients with a significant increase in gastric volume before extubation to avoid postoperative aspiration.

In this study, we found that older patients had a higher incidence of postoperative gastric volume increase by logistic regression analysis (*P* = 0.008, odds ratio 1.186, 95% *CI* 1.046–1.346). This may be because the esophageal wall of older patients is more compliant than that of younger patients, and gastric motility is reduced at an advanced age. Moreover, esophageal sphincter tone, barrier pressure between the esophagus and stomach, and intragastric pressure decreased significantly under general anesthesia, particularly in patients receiving muscle relaxants^1,18,19^. These physiological changes make it feasible to downflow large volumes of liquid during surgical procedures. In 2013, Perlas et al. found that the gastric volume decreased with advancing age based on ultrasound and endoscopy findings^15^. This finding seems to be inconsistent with our results. However, they used ultrasound to assess the volume of baseline gastric secretions in elective surgical patients but not fluid ingestion.

Gastroparesis has been confirmed to be an important factor in delaying gastric emptying and increases the risk of aspiration at anesthesia induction in surgical patients with diabetes mellitus, due to its secondary gastrointestinal autonomic dysfunction^20^. In this study, we found that postoperative gastric volume in diabetic patients was significantly higher than that in non-diabetic patients (*P* = 0.031, odds ratio 7.808, 95% *CI* 1.988–30.658), despite following preoperative fasting guidelines and a completely empty stomach before surgery. This surprising finding has rarely been reported in the literature. The clinical relationship between diabetes mellitus and its complications and intraoperative changes in gastric volume in patients undergoing EETS procedures is still unclear.

In addition, we found that longer surgical duration was also an independent risk factor associated with postoperative risk stomach (*P* = 0.005, odds ratio 1.028, 95% *CI* 1.008–1.048). This indicates that the volume ingested increased with prolonged surgical duration. However, there was no significant correlation between duration of anesthesia, type of anesthesia, ASA physical status classification, and intraoperative volume change.

The antral CSA measured by ultrasound is positively correlated with the volume of gastric contents, and the formula for volume calculation was formulated in a previous study^15^. We used a gastric fluid volume > 1.5 mL kg^−1^ to define the postoperative risk stomach, which is often used as the cutoff value to assess the aspiration risk at induction of anesthesia^16^. However, the minimum limit of gastric volume for increasing the risk of aspiration has not been well defined in adult surgical patients^21^. Moreover, the volume of fluid aspirated into the lungs is not necessarily equal to the intragastric volume, and the pathogenesis and pathophysiology differ between preoperative regurgitation and PONV^1,6,9,10^. Therefore, the critical value of gastric volume, which is specifically used to assess the postoperative aspiration risk, should be further investigated.

Our findings emphasize that bedside ultrasound can be used to quickly and reliably determine the gastric volume to assess the potential postoperative risk of aspiration in patients undergoing EETS. After orogastric suction under ultrasound guidance, gastric volume decreased significantly in our population, and no patients experienced aspiration at extubation. Throat packing could serve as a physical barrier that effectively prevents the downward flow of irrigation fluid and blood from the oropharynx to the esophagus and stomach^22^. However, throat packing can cause postoperative throat pain, mucosal injury, inflammation, and accidental airway obstruction^23,24^. Moreover, the application of throat packs in EETS has not been reported in the literature, and the effect of throat packs on postoperative gastric contents in patients undergoing EETA remains unclear. Therefore, at our institution, we do not recommend routine use of a throat pack during EETS.

This study has several limitations. First, our results may not reflect the actual incidence of intraoperative gastric volume changes because gastric emptying occurred continuously during the long surgical duration, particularly in the non-empty states. Second, we did not determine the baseline volume of gastric secretions during the EETS procedure. Therefore, we do not know the effect of autonomic gastric secretion on postoperative volume changes. Furthermore, because the number of patients with postoperative risk stomach is still small, we did not conduct a subgroup analysis to evaluate the effect of different types of functional pituitary adenomas on intraoperative gastric volume change. We also did not exclude patients with diabetes, gastroesophageal reflux, and patients using prokinetic drugs from our study, because these conditions may affect the gastric residual volume. Finally, our findings only reflect the local patterns related to intraoperative management (e.g., no throat packing) and the experience of the surgical teams. Larger sample sizes and multicenter studies are required to further assess the incidence and impact of gastric volume changes associated with endonasal transsphenoidal pituitary surgery in different patient populations.

## Conclusions

Our results showed a significant increase in gastric volume in some patients who underwent EETS. Bedside ultrasound measurements of gastric volume can be used to assess the postoperative aspiration risk, particularly in older diabetic patients with a longer surgical duration.

## Data Availability

The datasets used and/or analyzed during the current study are available from the corresponding author upon reasonable request.
